# Mapping Stripe Rust Resistance in a BrundageXCoda Winter Wheat Recombinant Inbred Line Population

**DOI:** 10.1371/journal.pone.0091758

**Published:** 2014-03-18

**Authors:** Austin J. Case, Yukiko Naruoka, Xianming Chen, Kimberly A. Garland-Campbell, Robert S. Zemetra, Arron H. Carter

**Affiliations:** 1 Department of Crop and Soil Science, Washington State University, Pullman, Washington, United States of America; 2 Wheat Genetics, Quality, Physiology, and Disease Research Unit, Agricultural Research Service, United States Department of Agriculture, Pullman, Washington, United States of America; 3 Department of Crop and Soil Science, Oregon State University, Corvallis, Oregon, United States of America; Oklahoma State University, United States of America

## Abstract

A recombinant inbred line (RIL) mapping population developed from a cross between winter wheat (*Triticum aestivum* L.) cultivars Coda and Brundage was evaluated for reaction to stripe rust (caused by *Puccinia striiformis* f. sp. *tritici*). Two hundred and sixty eight RIL from the population were evaluated in replicated field trials in a total of nine site-year locations in the U.S. Pacific Northwest. Seedling reaction to stripe rust races PST-100, PST-114 and PST-127 was also examined. A linkage map consisting of 2,391 polymorphic DNA markers was developed covering all chromosomes of wheat with the exception of 1D. Two QTL on chromosome 1B were associated with adult plant and seedling reaction and were the most significant QTL detected. Together these QTL reduced adult plant infection type from a score of seven to a score of two reduced disease severity by an average of 25% and provided protection against race PST-100, PST-114 and PST-127 in the seedling stage. The location of these QTL and the race specificity provided by them suggest that observed effects at this locus are due to a complementation of the previously known but defeated resistances of the cultivar Tres combining with that of Madsen (the two parent cultivars of Coda). Two additional QTL on chromosome 3B and one on 5B were associated with adult plant reaction only, and a single QTL on chromosome 5D was associated with seedling reaction to PST-114. Coda has been resistant to stripe rust since its release in 2000, indicating that combining multiple resistance genes for stripe rust provides durable resistance, especially when all-stage resistance genes are combined in a fashion to maximize the number of races they protect against. Identified molecular markers will allow for an efficient transfer of these genes into other cultivars, thereby continuing to provide excellent resistance to stripe rust.

## Introduction

Wheat (*Triticum* spp.) is a staple crop for more than one third of the world population [1]. Due to its intense cultivation, wheat has numerous disease challenges, the most destructive and wide spread of which are the rusts, caused by *Puccinia* spp. [2]. Wheat stripe rust (also known as yellow rust), caused by *Puccinia striiformis* Westend. f. sp. *tritici* Erikss. (*Pst*), is a destructive foliar disease affecting wheat production in many growing regions throughout the world [3]–[4]. In the United States (U.S.), stripe rust frequently causes severe damage west of the Rocky Mountains, but is a growing problem in other parts of the country [5]. Historically, the Pacific Northwest (PNW) region of the U.S. has had the most severe epidemics of stripe rust. In this region mild winters followed by cool wet springs and dry cool summers provide ideal conditions for stripe rust infection and survival [4]–[5].

Stripe rust is composed of diverse populations of many different races which exhibit a complex range of virulence and avirulence patterns when screened against a standard set of differential wheat genotypes [5]–[7]. The evolution of new races can rapidly render previously resistant cultivars susceptible, leading to severe and widespread epidemics. Such was the case of the stripe rust epidemics of the early 2000's when new races with virulence combinations (*Yr8* and *Yr9*) that had never previously been reported in the U.S. appeared, causing multimillion dollar losses [3], [8]. However, winter wheat losses were minimal because major cultivars grown in the PNW had durable non race-specific resistance and were not affected by new virulent races [3], [5], [6].

Resistance to stripe rust can be categorized into two basic types, all-stage resistance (also known as seedling resistance) and adult-plant resistance. All-stage resistance is typically race specific providing protection from only a defined set of races [5]. It is expressed at high levels throughout the plant's growth stages and is inherited qualitatively. This race specific interaction closely follows the gene-for-gene model described by Flor [9]. However, this form of resistance can be instable with the average effective life span of a single all-stage resistance gene of 3.5 years [4]–[5]. Currently the main all-stage resistance genes which are used in breeding programs and are effective against all currently identified races in the U.S. are *Yr5*, *Yr15* and *Yr45* [3], [8], [10]. Pyramiding multiple genes together can prolong the effective life span of all-stage resistance genes providing greater stability [8]. In contrast to all-stage resistance, adult-plant resistance is expressed primarily during later stages of plant growth and is usually a non-race specific resistance [5]. High-temperature adult-plant resistance (HTAP) is characterized by susceptible reactions during seedling stages under low temperatures followed by increased resistance as plants mature and weather becomes warm [5], [11]–[12]. HTAP resistance is globally distributed in many wheat cultivars and has been shown to provide durable resistance for more than 40 years [4], [13]–[14]. This type of resistance has been shown to be quantitatively inherited and there has been success in mapping and identifying quantitative trait loci (QTL) associated with HTAP resistance [15]–[20]. Identifying genetic markers linked to stripe rust resistance genes greatly enhances the ability of resistance to be quickly introgressed into breeding material using marker-assisted selection [4].

The soft white winter wheat (*T. aestivum* L.) mapping population created from the cross ‘Brundage’ (PI 599193) by ‘Coda’ (PI 594372) at the University of Idaho has been used to map several traits including *Pch1* resistance to eyespot caused by *Oculimacula yallundae* [21], Cephalosporium stripe caused by *Cephalosporium gramineum* [22], and the *compactum* locus [23]. The population also segregates for resistance to stripe rust. Therefore, we hypothesized that Coda carries a high level of heritable resistance to stripe rust and this resistance is likely conferred by one or more genes, which can be identified by QTL mapping. The objective of this study was to map QTL for stripe rust resistance and determine the relationship between identified QTL and known genes for stripe rust resistance. In the following work we report the successful identification of several stripe rust resistance QTL and hypothesize their origin and implications in breeding for rust resistance.

## Materials and Methods

### Ethics Statement

No permits were necessary to conduct reported field experiments. Stripe rust is a naturally occurring plant pathogen in the reported environments. No exotic cultures of the pathogen were use and as such permits were not required to conduct described research. Research was conducted on land owned by Washington State University or by the University of Idaho. No protected species were sampled. No animal subjects were used in described research. All experiments reported in this manuscript comply with all federal, state and university rules and regulations.

### Plant material

Brundage is a soft white winter wheat cultivar adapted to the growing conditions of the PNW with the pedigree ‘Stephens’ (CItr 17596)/‘Geneva’ (PI 505819) [24]. Brundage is weakly resistant to stripe rust. ‘Brundage 96’ (PI 631486) is a reselection out of Brundage for better stripe rust resistance [25]. The recombinant inbred line (RIL) population was developed using the original Brundage. Coda is a soft white winter club wheat cultivar, with pedigree ‘Tres’ (CItr 17917//‘Madsen’ (PI511673)/‘Tres’ [26]. Coda is adapted to growing conditions in the PNW and has strong stripe rust resistance. This RIL population was created at the University of Idaho where, after the initial cross was made, the F_1_ was allowed to self-pollinate and then single-seed descent carried the population to the F_6:7_ generation, from which 268 lines were derived [21]–[23]. Included in this trial were both parents and the stripe rust susceptible check WA7821. Parental seed material for this experiment was derived from the original plants used to create the population. Brundage and Coda are both heterogeneous cultivars, as they were selected from mid-generation headrows. For example, the row that was selected for the cultivar Coda was heterozygous for the *Pch1* gene, and heterogeneity of plants exists within the cultivar of either carrying the *Pch1* gene or not. Therefore, care was taken to ensure that the Brundage and Coda seed used in crossing to develop the population was maintained, and only seed from the original parental lines were used for any future analysis of the parents.

### Stripe rust screening

Stripe rust evaluation was conducted in a total of nine site-year locations: Whitlow Farm near Pullman, WA in 2006 (wl06) and 2010 (wl10), Mount Vernon, WA in 2006 (mv06) and 2010 (mv10), the Parker Farm, near Moscow, ID in 2010 (ui10) and 2011 (ui11), the Spillman Agronomy Farm near Pullman, WA in 2010 (pu10) and 2011 (pu11), and Central Ferry Farm in south-central Washington in 2010 (cf10). The locations in Pullman, WA; Moscow, ID; and Mount Vernon, WA are rain fed locations and Central Ferry, WA is irrigated. Mount Vernon, WA is located in the high rainfall area west of the Cascade Mountain range whereas the others are in the semi-arid wheat producing areas east of the Cascade Mountain range. Plots were sown in the fall and maintained according to common commercial winter wheat production practices of the region. Plots at Spillman Agronomy Farm and at Central Ferry Farm consisted of five grams of seed sown into 1 m-rows spaced 35 cm apart. Hand planted seed hills of five grams of seed per hill spaced 30 cm apart were evaluated at Whitlow Farm, Mount Vernon, and Parker Farm. Trials were designed as a randomized complete block with three replications per trial except for wl06 and mv06 which were unreplicated and pu11 which had only two replicates.

Stripe rust evaluation was conducted under naturally occurring stripe rust infection. The susceptible winter wheat line WA7821 was used as an inoculum spreader and was planted evenly every 20 plots throughout the trials as well as surrounding them to ensure uniform stripe rust infection. The population was scored for stripe rust using infection type (IT) rating, scale of zero to nine [27], and disease severity (DS) rating of percent leaf area in the row infected, using the modified Cobb Scale as suggested by Peterson et al. [28]. Data was collected every three to seven days based on disease progression. Disease rating evaluation started when DS became relatively uniform on the susceptible check WA7821 and continued until active infection was no longer observed.

Race specificity at the seedling stage was investigated by inoculating 268 RILs and parental lines in the greenhouse with stripe rust races PST-100, PST-114 and PST-127 [6]. Plants were planted in 96 well trays filled with #1 Sunshine Mix (Sun Gro Horticulture, Bellevue, WA). Seedlings were inoculated at the three to five leaf stage with urediniospores suspended in isoparaffin oil. After inoculation plants were placed in a dew chamber (Percival Scientific, Inc, Perry, IA) at 100% humidity in the dark for 24 h at 10°C. Plants were then moved to a growth room set at a diurnal temperature cycle of 4°C at night and 20°C at day with a 16 h photoperiod [29]. At 20 days after inoculation the plants were scored based on IT rating scale as described above, where a score of three or less was considered resistant.

Seedling and adult plant greenhouse tests performed by Dr. Chen rated Coda as resistant to all races tested as both a seedling and adult-plant (Dr. Xianming Chen personal communication, December 4, 2012). Races tested included PST-37, PST-45, PST-100, PST-114, PST-116, and PST-127, which collectively represent virulence to differentials one through twenty, with the exception of differential seven possessing *Yr5* [6]. In the same tests Brundage 96, a derivative of Brundage, was rated as resistant or moderately resistant to PST-45 and intermediate to PST-114.

### Data analysis

For both IT and DS, data were analyzed based on the reading of the individual RIL, averaged over replicates, when both the susceptible check and parental lines were showing uniform symptoms throughout the trial. Mean (average within a location) and grand mean (average over all locations) DS and IT values were calculated for all RILs and parents. Statistical analysis of variance of IT and DS mean values was conducted within each environment using SAS v9.1 (SAS Institute, Raleigh, NC). The Proc GLM procedure was used to test the genotype effect as well as replication by genotype interaction. Residuals were normally distributed and apart from skewness, the data fits the assumptions for the method of analysis. SAS code provided by Holland et al. [30] was used to calculate the broad-sense heritability (H^2^), using the formula H^2^ = Var(G)/Var(P) (where Var(G) is the genotypic variance and Var(P) is phenotypic variance). Because this population was inbred, the broad sense heritability consists mainly of additive and epistatic effects and provides an upper limit to narrow sense heritability. Broad-sense heritability was calculated for each location for IT and DS separately.

### Genotyping

The Brundage by Coda population has previously been genotyped using SSR (simple sequence repeat; *Xbarc*, *Xpsp*, *Xwmc*, *Xgwm*, *Xgdm*) and DArT (Diversity Array Technology; *wPt*) genetic markers as described in Quincke et al. [22], Johnson et al. [23] and Leonard et al. [21]. Single nucleotide polymorphism (SNP) markers (*Xiwa*) were evaluated on the population using the Illumina GoldenGate assay as described by Akhunov et al. [31] and Cavanagh et al. [32]. A total of 1,984 polymorphic SNP markers were identified.

An initial QTL analysis (see below for procedure) identified significant regions of the genome that were associated with stripe rust resistance. A search on GrainGenes (http://wheat.pw.usda.gov) for additional SSR markers that have been previously determined to be in regions of the genome that were of interest was conducted. These markers were then screened on the population using the following conditions. Genomic DNA was extracted from 30–50 mg of fresh leaf tissue as described by Riera-Lizarazu et al. [33]. Marker sequences and annealing PCR temperatures were used as recommended on GrainGenes. PCR primers were synthesized to include the M13-tail on the forward primer [34]. The reaction mixture was a total of 12 µl consisting of 120 ng of template DNA, 1.2 µl 10× Mg-free PCR buffer (New England Biolabs, Ipswich, MA), 1.0 Uof *Taq* DNA polymerase, 1.0 mM of MgCl_2_ (Fermentas, Glen Burnie, MD), 200 µM dNTP (New England Biolabs, Ipswich, MA), 0.05 µM M13-tailed forward primer, 0.25 µM reverse primer, and 0.20 µM appropriate M13-tailed fluorophore for the use with the ABI 3730×l DNA Analyzer (Applied Biosystems, Foster City, CA). PCR amplification conditions were as follows: 5 min initial denaturation at 94°C followed by 42 cycles of 1 min of denaturation at 94°C, 1 min of annealing at 50–65°C (according to primer Tm) and 1 min extension at 72°C, with a final extension step of 10 min at 72°C.

### Map construction and QTL analysis

A chi-squared test was performed to test for segregation distortion of markers with an expected ratio of 1∶1. Linkage map construction was performed using Join Map v4.0 [35] and maximum likelihood mapping was performed to order linked markers. Linkage groupings were created using the “Create Groups Using the Groupings Tree” tool and linkage groups were selected based on a minimum LOD score of 4. Marker distances were calculated using default estimation parameters described in Van Ooijen [35]. Linkage group identity and order were assigned based on evaluation of similarities to published linkage maps available in GrainGenes (http://wheat.pw.usda.gov) and the SNP consensus map of Cavanagh et al. [32]. Map distances were calculated in cM units with the Haldane mapping estimation function [36], which was then recalculated to Kosambi [37] units once imported into WinQTLCart v2.5 [38]–[39].

QTL analysis was undertaken with WinQTLCart v2.5 software. Composite interval mapping [40]–[41] was performed with forward and backward stepwise regression to identify QTL, using a window size of 10 cM, a probability in and out of 0.1, five control markers and a walk speed of 1 cM. QTL were identified for the mean IT and DS values within each environment and for the IT and DS values calculated over environments. Seedling reaction data was imported as binary data for RIL IT as either 1 resistant or 8 susceptible. Significant LOD threshold values were defined using a permutation test with 1,000 permutations. QTL positions were identified based on peak LOD score for grand mean DS values and IT position was based on average peak position and peak LOD scores for individual locations at that position. QTL confidence intervals were established based on a one-LOD drop off from the QTL peak position [42].

Linkage map figures were generated using Map Chart v2.2 [43]. WinQTLCart v2.5 provided parental effect calculations and R^2^ values. The two major loci controlling seedling resistance on chromosome 1B had an effect on the significance of other markers for adult plant resistance. In order to control for these large seedling resistance effects, mixed model analysis was performed using SAS Proc Mixed for each previously identified significant marker. In these models, the markers *Xiwa7298* at 1B.1 and *Xiwa7480* at 1B.2 were considered to be fixed because they were consistently the markers in the interval that had the greatest effect for seedling resistance for both IT and DS scores. The reduced model included the only fixed effect markers *Xiwa7298* and *Xiwa7480* while the full model included those fixed effects plus a random effect for a single different marker. Adjusted R^2^ values were calculated for each marker as:
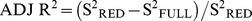
where S^2^_RED_ = the residual variance from the reduced model and S^2^_FULL_ = the residual variance from the he full model. A Tukey's mean separation test of IT and DS mean values was performed on RILs grouped according to which QTL combination they carried using SAS Proc GLM means Tukey procedure.

## Results

### Stripe rust evaluation

**Figure 1:**
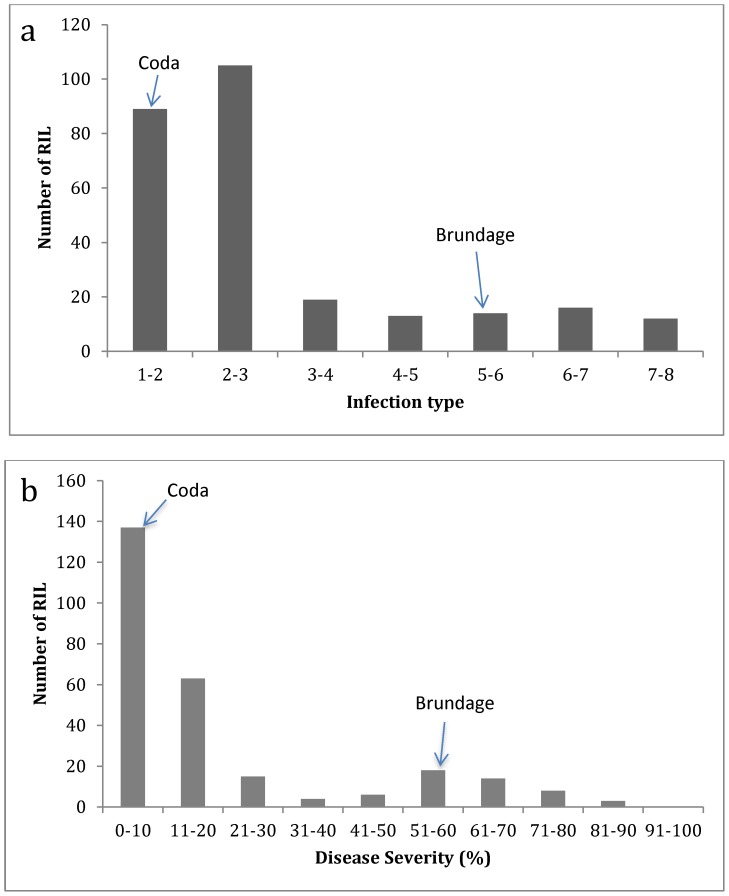
Distribution of infection in the Brundage by Coda RIL population. Distribution of: (**a**) infection type (IT) and (**b**) disease severity (DS) grand mean (averaged over all locations) values. Arrows indicate resistant parent (Coda) and susceptible parent (Brundage) score groups.

Significant stripe rust infection was observed in the field at all locations. The IT and DS values of the stripe rust susceptible spreader line WA7821 ranged from six to eight and 40 to 100%, respectively. The resistant parent Coda was observed to have lower scores compared to the susceptible parent Brundage at all locations, except for ui11 where they had similar scores. At all locations the mean of IT (except ui11) and DS values for all RILs were greater than that of the resistant parent Coda (Figure S1). A significant genotype effect was observed (*P*<0.0001) for both IT and DS at all locations (Table S1 and S2). The distribution of RIL grand mean IT and DS values for field data are skewed toward resistant (Figure 1). IT and DS mean values were strongly correlated (R^2^ = 0.88); however, the distribution of DS values was more skewed toward resistant than that of IT. Broad sense heritability of IT mean values for all locations ranged from 0.54 to 0.81 and 0.49 to 0.92 for DS mean values (Table S1 and S2). Trials in 2006 were unreplicated and therefore heritability estimates were not possible. The most predominate races in 2006 in Mount Vernon were PST-25 followed by PST-100; in Pullman the population was equally represented by PST-100 and PST-115 [6]. In 2010, the most common race in Mt Vernon was PST-35 whereas in Eastern Washington it was predominantly PST-139/PST-127 with some PST-137. In 2011 the most prevalent race in Pullman was PST-143 (http://striperust.wsu.edu/races/stripe-rust-race-data.html).

### Genetic linkage map construction

The population was genotyped with a total of 2,391 polymorphic DNA markers including 1,984 SNP, 232 DArT and 175 SSR markers. A total of 2,144 markers were mapped to 32 discrete linkage groups (significant linkage groups are presented in Figure S2). The map had a total map length of 3,294.2 cM with an average interval distance of 1.54 cM. These 32 linkage groups were identified based on homology to published maps using a GrainGenes search and comparison to a consensus map developed by Cavanagh et al [32]. All chromosomes, with the exception of 1D, were represented with one or more linkage groups. The average linkage group length was 102.9 cM. These 32 groups were used in whole genome QTL scans.

### QTL analysis

**Figure 2:**
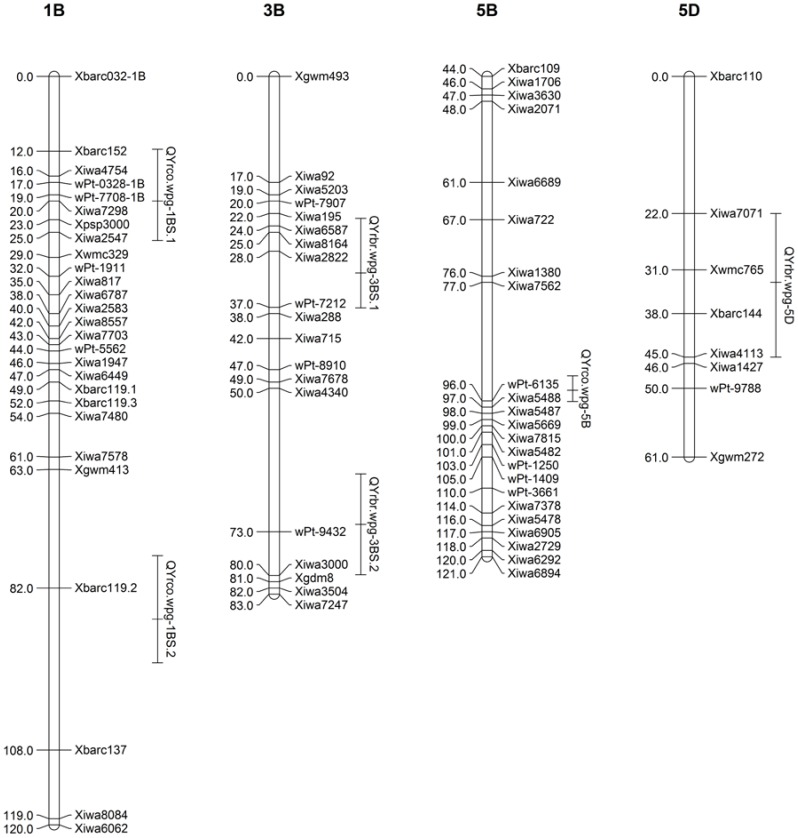
Selected portions of the genetic linkage map from the Brundage by Coda RIL population. Selected portions of the genetic linkage map from the Brundage by Coda recombinant inbred line population showing quantitative trait loci (QTL) containing portions of chromosomes 1B, 3B, 5B and 5D. Solid bars to the right of linkage maps outline QTL positions for infection type values and disease severity values, with a dash marking the position of the peak LOD score. Locations include: Central Ferry, WA; Mount Vernon, WA; Spillman Farm Pullman, WA; Whitlow Farm Pullman, WA; Parker Farm Moscow, ID; growth chamber seedling tests with races: PST-100, PST-114 and PST-127. Dates include 2006, 2010, and 2011.

Composite interval mapping was used to scan the genome for QTL associated with IT and DS mean values for each location as well as the grand mean. QTL were identified on chromosomes 1B, 3B, 5B and 5D (Table 1). A major QTL was found on 1B that was significantly associated with mean IT and DS mean values in all locations as well as in seedling reaction to race PST-100 (LOD 16.0) and PST-127 (LOD 48.6) (Table 1; Figure 2). The resistant allele was inherited from Coda and was given the designation *QYrco.wpg-1B.1* (Coda designated as co). This QTL accounts for 50% of the phenotypic variation when averaged over all field locations and decreased IT values by 26%. A second significant Coda derived QTL on chromosome 1B was also detected in three field locations and in seedling reaction to race PST-100 and PST-114 and accounts for an average of 12% of the phenotypic variation. This QTL was named *QYrco.wpg-1B.2* and appears to be race specific. Two QTL were detected on chromosome 3B in response to IT and DS mean values. The resistant alleles for the 3B QTL were inherited from Brundage and designated *QYrbr.wpg-3B.1* and *QYrbr.wpg-3B.2* (Brundage designated as br) (Figure 2). *QYrbr.wpg-3B.1* was found in every location in response to both IT values and DS values (with the exception of Moscow field trials) whereas *QYrbr.wpg-3B.2* was never detected in 2011 or in Central Ferry in 2010. Averaged over all locations, *QYrbr.wpg-3B.1* had a LOD score of 11.0, but only accounted for 5% of the phenotypic variation. Adjusted R^2^ values showed a similar value of 5%. Similarly, *QYrbr.wpg-3B.2* only accounted for 2 and 3% of the phenotypic variation for IT and DS, and had a LOD score of 18.2 and 20.2, respectively. Neither of the 3B QTL was detected in seedling reactions, indicating they are adult plant resistance QTL with weak avirulence to current field race populations. One Coda derived QTL was found on chromosome 5B and as was given the designation *QYrco.wpg-5B*. This QTL was detected in response to IT mean values in all locations except for Pullman in 2011 and DS mean values at all locations except for Moscow and Pullman in 2011. *QYrco.wpg-5B*, averaged over all field locations, had a LOD value of 17.5, and only accounted for 3.4% of the phenotypic variation, probably due to the large effect of the 1B QTL. When taking into account the 1B QTL, the phenotypic variation accounted for by this locus only increased to 4.3%. Additionally, the adjusted R^2^ values indicated that the two Mount Vernon locations were no longer significant for IT values (Table 1). This is probably due to the unique race structure of Mount Vernon, which is different than the other tested locations. Interestingly though, these locations were still significant for DS, whereas the Whitlow and Moscow 2010 were no longer significant for DS mean values. *QYrco.wpg-5B* was not detected in seedling reaction and appears to be an adult plant resistance QTL. A final QTL was detected on chromosome 5D for seedling reaction to PST-114 only. This QTL was named *QYrbr.wpg-5D* and appears to be a seedling resistance gene. This QTL had a LOD score of 7.9 and accounted for 12% of the phenotypic variation.

**Table 1:** Significant quantitative trait loci for stripe rust resistance identified by composite interval mapping in the Coda by Brundage recombinant inbred line mapping population.

QTL	Location^a^	Chr	LOD	Linked Markers	Peak Position	Interval^b^	R^2^ (%)^c^	Adjusted R^2^ (%)^d^	Parent
Seedling IT									
*QYrco.wpg-1B.1*	Pst-100	1B	16.0	*Xiwa4754*, *Xiwa7298*, *Xpsp3000*, *Xiwa2547*	17.0 cM	12.4–26.3	24		Coda
	Pst-127		48.6		23.4 cM	22.6–27.8	56		Coda
*QYrco.wpg-1B.2*	Pst-100	1B	9.6	*Xbarc119*, *Xbarc137*	87.8 cM	80.9–95.3	14		Coda
	Pst-114		30.0		92.0 cM	82.3–94.0	06		Coda
*QYrbr.wpg-5D*	Pst-114	5D	7.9	*Xwmc765*, *Xbarc144*	33.5 cM	27.5–37.3	12		Brundage
Adult Plant IT									
*QYrco.wpg-1B.1*	pu11	1B	52.5	*Xiwa4754*, *Xiwa7298*, *Xpsp3000*, *Xiwa2547*	14.2 cM	11.2–21.4	72		Coda
	mv10		44.2		20.0 cM	18.0–21.1	51		Coda
	mv06		76.3		20.1 cM	18.0–21.2	73		Coda
	wl10		35.1		20.1 cM	16.0–21.1	4		Coda
	cf10		28.3		24.2 cM	21.3–27.3	29		Coda
	ui10		61.5		24.3 cM	21.1–27.2	64		Coda
	pu10		70.1		24.3 cM	21.4–26.7	87		Coda
	ui11		36.2		24.3 cM	21.4–27.3	51		Coda
	wl06		17.4		25.3 cM	21.4–26.6	22		Coda
*QYrco.wpg-1B.2*	ui10	1B	17.3	*Xbarc119*, *Xbarc137*	78.0 cM	77.0–78.7	14		Coda
	mv10		29.4		78.9 cM	76.8–83.7	11		Coda
	wl06		31.9		79.9 cM	77.9–82.9	12		Coda
*QYrbr.wpg-3B.1*	ui11	3B	6.3	*Xiwa195*, *Xiwa6092*, *Xiwa4725*, *Xiwa6587*, *Xiwa1702*, *Xiwa5201*, *Xiwa8164*, *Xiwa2822*, *Xiwa2908*, *Xiwa5426*, *Xiwa288*, *Xiwa289*, *Xiwa2493*, *Xiwa715*	23.8 cM	22.6–37.0	7	6	Brundage
	pu11		11		24.3 cM	16.1–27.7	8	9	Brundage
	cf10		9		31.7 cM	20.5–37.1	6	9	Brundage
	wl06		23.6		33.6 cM	27.8–37.1	4	1	Brundage
	ui10		16.6		34.7 cM	33.0–37.0	2	1	Brundage
	mv10		12.1		36.8 cM	25.0–43.0	3	4	Brundage
	pu10		6		37.3 cM	24.5–47.3	4	6	Brundage
	mv06		9.2		38.0 cM	24.0–45.0	2	5	Brundage
	wl10		5.6		43.6 cM	27.3–48.7	4	6	Brundage
*QYrbr.wpg-3B.2*	wl06	3B	31.2	*wPt-9432*, *Xiwa3000*, *Xiwa6213*, *Xiwa2999*, *Xgdm8*	70.6 cM	63.7–75.1	1	1	Brundage
	pu10		14.8		70.7 cM	69.7–74.8	2	2	Brundage
	ui10		23.7		71.4 cM	67.4–75.5	2	1	Brundage
	wl10		8.1		72.5 cM	68.6–73.5	3	2	Brundage
	mv10		26.5		72.7 cM	71.8–79.9	1	-e	Brundage
	mv06		4.6		73.5 cM	72.5–74.4	2	1	Brundage
*QYrco.wpg-5B*	wl06	5B	34.1	*Xiwa5488*, *Xiwa2335*, *Xiwa2697*, *Xiwa7776*, *Xiwa6068*, *Xiwa3044*, *Xiwa7471*, *Xiwa5487*, *Xgwm639*	92.0 cM	78.3–95.8	1	1	Coda
	mv06		24.2		92.1 cM	91.3–96.2	1	-	Coda
	mv10		30.2		93.1 cM	89.2–95.9	1	-	Coda
	wl10		12.3		93.1 cM	90.2–96.7	6	6	Coda
	pu10		17.6		94.2 cM	91.1–96.9	6	4	Coda
	ui11		4.5		94.3 cM	93.0–94.5	2	3	Coda
	ui10		6.7		97.1 cM	78.8–110.4	5	9	Coda
	cf10		9.5		98.5 cM	91.7–100.4	5	12	Coda
Adult Plant DS									
*QYrco.wpg-1B.1*	wl10	1B	61.1	*Xiwa4754*, *Xiwa7298*, *Xpsp3000*, *Xiwa2547*	13.4 cM	11.3–21.4	69		Coda
	pu10		90.3		13.6 cM	11.3–21.4	83		Coda
	cf10		49.3		14.3 cM	11.3–21.3	44		Coda
	pu11		48.1		14.3 cM	11.3–21.4	68		Coda
	wl06		38.6		16.9 cM	15.9–17.9	32		Coda
	ui10		40.1		21.0 cM	11.1–21.5	57		Coda
	mv06		64.8		21.1 cM	11.3–21.4	58		Coda
	mv10		50.7		22.4 cM	11.3–21.4	64		Coda
	ui11		58.3		24.3 cM	21.1–27.2	64		Coda
*QYrco.wpg-1B.2*	wl06	1B	36.1	*Xbarc119*, *Xbarc137*	77.9 cM	76.8–87.3	1		Brundage
	mv06		37.0		78.9 cM	76.9–83.9	2		Brundage
	mv10		19.0		79.0 cM	76.8–84.8	2		Brundage
*QYrbr.wpg-3B.1*	pu11	3B	5.4	*Xiwa195*, *Xiwa6092*, *Xiwa4725*, *Xiwa6587*, *Xiwa1702*, *Xiwa5201*, *Xiwa8164*, *Xiwa2822*, *Xiwa2908*, *Xiwa5426*, *Xiwa288*. *Xiwa289*, *Xiwa2493*, *Xiwa715*	28.7 cM	27.3–36.8	4	6	Brundage
	pu10		10.6		28.9 cM	26.1–29.8	2	-	Brundage
	cf10		4.7		28.9 cM	23.8–29.8	3	2	Brundage
	wl06		33.5		35.5 cM	31.7–37.1	6	2	Brundage
	wl10		16.7		36.8 cM	35.7–37.3	2	3	Brundage
	mv06		29.1		37.0 cM	35.5–37.3	2	6	Brundage
	mv10		12.4		37.0 cM	35.7–38.2	3	4	Brundage
*QYrbr.wpg-3B.2*	pu10	3B	5.0	*wPt-9432*, *Xiwa3000*, *Xiwa6213*, *Xiwa2999*, *Xgdm8*	71.4 cM	70.6–76.9	2	-	Brundage
	wl10		23.6		71.6 cM	70.4–73.5	2	3	Brundage
	wl06		36.1		72.3 cM	69.3–74.1	6	1	Brundage
	ui10		3.4		72.5 cM	67.6–73.5	1	1	Brundage
	mv06		35.8		72.7 cM	71.4–76.7	2	2	Brundage
	mv10		17.5		75.5 cM	73.5–76.4	3	2	Brundage
*QYrco.wpg-5B*	wl10	5B	23.2	*Xiwa5488*, *Xiwa2335*, *Xiwa2697*, *Xiwa7776*, *Xiwa6068*, *Xiwa3044*, *Xiwa7471*, *Xiwa5487*, *Xgwm639*	93.0 cM	91.2–96.0	2	-f	Coda
	pu10		7.7		93.1 cM	92.1–96.8	3	5	Coda
	mv10		19.9		94.1 cM	89.3–95.9	1	1	Coda
	mv06		36.8		94.2 cM	89.4–95.9	1	6	Coda
	wl06		34.0		95.3 cM	94.3–97.1	1	2	Coda
	ui10		12.6		98.8 cM	95.8–100.3	1	-	Coda
	cf10		7.8		100.4 cM	93.7–100.6	4	13	Coda

a Seedling data was collected under controlled room environment for the three races identified. Adult plant resistance was rated under natural inoculation in field conditions for: Mount Vernon, WA (mv), Pullman, WA (pu), Central Ferry, WA (cf) Whitlow Farm, Pullman, WA (wl), University of Idaho Farm, Moscow, ID (ui) for the years 2006 (06), 2010 (10) and 2011 (11).

b QTL interval based on a one LOD drop-off.

c R^2^ values for each interval calculated based on composite interval mapping in QTL Cartographer.

d R^2^ values adjusted for the effects of the major seedling genes at *Xiwa7298* and *Xiwa7480*, the most significant markers for seedling resistance at *QYrco.wpg-1B.2* and *QYrco.wpg-1B.2*. R^2^ values were calculated using mixed model analysis with *Xiwa7298* and *Xiwa7480* held fixed and each other markers considered random. R^2^ values were then averaged over markers in each interval.

e Dash marks indicate the location was no longer significantly associated with stripe rust resistance using the adjusted R^2^ values.

f The adjusted R^2^ for linkage group *QYrco.wpg-5B* is based on marker *Xgwm639* only as other markers had a high amount of missing data.

Linkage of QTL with SSR markers mapped by physical mapping using deletion bins places QTL on chromosome 1B on the short arm (bin C-1BS10-0.50), and QTL on 3B (bin C-3BL2-0.22), 5B (bin 5BL1-0.55-0.75) and 5D (bin c-5DL1-0.60) on the long arm [44]. Haplotype analysis suggested that *QYrco.wpg-1B.1* is Coda derived and shared a haplotype with Tres whereas *QYrco.wpg-1B.2* is also Coda derived but haplotype analysis indicates it is derived from Madsen. Data was not available for haplotype analysis of *QYrbr.wpg-5D*. *QYrbr.wpg.3B.1* and *QYrbr.wpg-3B.2* are Brundage derived and shared a haplotype with Stephens. *QYrbr.wpg-5B* is Coda derived and shared a haplotype with both Madsen and Coda.

### Effect of QTL

To assess how different QTL and QTL combinations affect the adult plant IT and DS values for the population, each RIL within the population was categorized based on its QTL complement (Table 2). RILs with no QTL for stripe rust resistance were found to have higher IT and DS values than the resistant parent Coda, but not the susceptible parent Brundage. Tukeys mean separation test showed significant differences (*P*<0.05) between QTL groupings. *QYrbr.wpg*-5D was only found in reaction to seedling infection and therefore was excluded from this analysis. Lines carrying *QYrco.wpg-1B.1* in combination with any other QTL (except *QYrbr.wpg.3B.1*) were not significantly different from each other, but were significantly different than *QYrco.wpg-1B.1*. Interestingly, *QYrco.wpg-1B.1* in combination with *QYrbr.wpg.3B.1* demonstrated a 16 and 36% increase in IT severity and DS percentage, respectively. Additionally, *QYrbr.wpg.3B.1* with *QYrco.wpg-1B.2* or *QYrco.wpg-5B* had the highest IT and DS mean values, and were significantly higher than with either *QYrco.wpg-1B.2* or *QYrco.wpg-5B* alone. It appears that *QYrbr.wpg.3B.1*in combination individually with other genes actually increase the mean severity values of those lines. The exception is lines carrying both *QYrbr.wpg.3B.1* and *QYrbr.wpg.3B.2*, which had lower mean IT and DS values than *QYrbr.wpg.3B.2* alone. Unfortunately, no RIL were identified which carried only the *QYrbr.wpg.3B.1* locus, therefore comparisons to this locus alone could not be done. The addition of *QYrco.wpg-1B.1* with two or more adult plant resistance QTL, demonstrated the lowest IT values of the group. *QYrco.wpg-1B.2*, *QYrbr.wpg-3B.2*, and *QYrco.wpg-5B* alone had moderately high mean IT values of 5.5, 5.7, and 6.4 and mean DS values of 44, 53, and 58%, respectively. This further indicates *QYrco.wpg-1B.2* has limited effectiveness under field conditions and the low phenotypic variation explained by *QYrbr.wpg-3B.2* and *QYrco.wpg-5B*.

**Table 2:** Grand mean, averaged over all field locations for infection type (IT) values (**a**) and disease severity (DS) values (**b**) for recombinant inbred line (RIL) possessing different QTL for stripe rust resistance.

QTL Combination	Average IT value	Grouping	QTL Combination	Average DS value (%)	Grouping
1B1, 3B2, 5B	1.5	A	Coda	5	A
1B1, 3B1, 3B2, 5B	1.7	A	1B1, 5B	6	A
1B1, 3B1, 5B	1.8	A	1B1, 1B2, 3B1, 3B2, 5B	6	A
1B1, 1B2, 3B1, 3B2, 5B	1.9	A	1B1, 3B1, 5B	6	A
1B1, 1B2, 3B1, 5B	1.9	A	1B1, 1B2, 3B1, 5B	7	A
Coda	2.0	AB	1B1, 3B1, 3B2, 5B	7	AB
1B1, 5B	2.2	AB	1B1, 3B2, 5B	8	AB
1B1, 1B2, 3B1, 3B2	2.2	AB	1B1, 1B2, 3B1, 3B2	9	AB
1B1, 1B2, 3B1	2.3	AB	1B1, 1B2, 3B1	9	AB
1B2, 3B1, 3B2, 5B	2.3	AB	1B1, 1B2	11	AB
1B1, 1B2, 3B2, 5B	2.4	AB	1B1, 3B1, 3B2	11	AB
1B1, 1B2, 5B	2.5	AB	1B1, 1B2, 3B2, 5B	12	AB
1B1, 1B2	2.5	AB	1B1, 1B2, 3B2	12	AB
1B1, 1B2, 3B2	2.7	AB	1B1, 1B2, 5B	12	AB
1B1, 3B1, 3B2	2.7	AB	1B2, 3B1, 3B2, 5B	12	AB
1B1, 3B2	2.9	ABC	1B1, 3B2	16	B
1B2, 5B	3.4	BCD	1B2, 5B	28	C
3B1, 3B2	4.0	CDE	1B1	34	CD
3B1, 3B2, 5B	4.4	CDEF	3B1, 3B2, 5B	36	DE
1B1	4.7	DEFG	1B2, 3B2, 5B	37	DE
1B2, 3B2, 5B	5.0	EFGH	3B1, 3B2	39	DE
1B2	5.5	EFGH	1B2	44	EF
1B1, 3B1	5.6	FGH	Brundage	50	FG
5B	5.7	FGH	1B1, 3B1	53	G
Brundage	6.0	GHI	5B	53	G
3B2, 5B	6.0	GHI	3B2, 5B	56	G
None	6.3	HIJ	3B2	58	G
3B2	6.4	HIJ	None	59	G
3B1, 5B	7.2	IJ	3B1, 5B	70	H
1B2, 3B1	7.6	J	1B2, 3B1	73	H

Letters above columns indicate Tukey's mean separation groupings of mean (averaged over all reps within a location) IT and DS values for RIL possessing different QTL for stripe rust resistance. QTL symbols are: *QYrco.wpg-1B.1* (1B1); *QYrco.wpg-1B.2* (1B2); *QYrbr.wpg-3B.1* (3B1); *QYrbr.wpg-3B.2* (3B2); *QYrco.wpg-5B* (5B); no resistance QTL (None); the susceptible parent (Brundage); and the resistant parent (Coda).

## Discussion

This study successfully mapped QTL for stripe rust resistance on chromosomes 1B, 3B, 5B and 5D, named *QYrco.wpg-1B.1*, *QYrco.wpg-1B.2*, *QYrbr.wpg-3B.1*, *QYrbr.wpg-3B.2*, *QYrco.wpg-5B*, *QYrbr.wpg-5D*. The most significant QTL detected was *QYrco.wpg-1B.1* which was detected in all locations for IT and DS as well as conferred resistance to PST-100 and PST-127 at the seedling stage. When evaluated together with *QYrco.wpg-1B.2* it also reduced IT values from a score of six to a score of two, and reduced DS in severity by 48% as compared to fully susceptible RILs (Table 2). Other QTL present on 3B and 5B were found to be adult plant associated QTL. Of these, *QYrco.wpg-5B* was the most significant and was found in all but one location for IT and two for DS. RILs carrying *QYrco.wpg-5B* or any 3B QTL in combination with *QYrco.wpg-1B.1* were observed to have similar scores, which were higher than RILs carrying 1B QTL only (Table 2). RILs containing all QTL in combination had lower scores for IT and DS than that of any 1B alone, demonstrating effectiveness of pyramiding adult plant and all-stage resistance genes.

The parents of Coda, Madsen and Tres, are both resistant as seedlings to PST-100 but differ in their reaction to PST-114 and PST-127, where Madsen is resistant to PST-114 and Tres is resistant to PST-127. *QYrco.wpg-1B.1* was found in reaction to PST-100 and PST-127, whereas *QYrco.wpg-1B.2* was found in reaction to PST-100 and PST-114 in the seedling stage. This suggests that *QYrco.wpg-1B.1* was derived from Tres and *QYrco.wpg-1B.2* was derived from Madsen. Furthermore, Coda shares a similar haplotype with Tres at *QYrco.wpg-1B.1* whereas Coda shares a similar haplotype with Madsen at *QYrco.wpg-1B.2*. Localization of QTL for seedling reaction with adult plant reaction suggests that *QYrco.wpg-1B.2* is a race specific all-stage resistance gene, whereas *QYrco.wpg-1B.1* has race specificity in the seedling stage but also very strong adult plant resistance to mixed race field populations. Further work needs to be done to confirm if this one locus is conferring both race-specific all-stage resistance and non-race specific adult plant resistance, or if these are two different loci tightly linked. Tres carries *YrTr1* and *YrTr2*, which have mapped to 6D and 3A respectively by monosomic analysis [45]. The resistance genes in Madsen have not been genetically mapped or characterized, although it is known to carry all-stage resistance genes and has high levels of adult plant resistance (Dr. Xianming Chen personal communication, December 4, 2012).

Numerous stripe rust genes have been identified on chromosome 1B including: *Yr3a,b,c* [46] *Yr21* [47], *Yr10* [48], *Yr15* [49] and *Yr24/26/CH42* [50], all located on 1BS. *Yr9* [51] and *Yr29* [52] are located on the 1RS of the 1BL.1RS chromosome and 1BL, respectively (reviewed by [53]). Several temporarily designated *Yr* genes have been mapped to 1B including: *YrAlp* [16] and *YrC142* [54] on 1BS and *YrCN17* [55], *YrExp1* [17] and *YrR212* [55] on 1BL. Furthermore several stripe rust resistance QTL have been mapped to chromosome 1B including: *QYr.sun-1B* [56], *QPST.jic-1BL* [57] and a QTL linked to *Xgwm259* [58].

Of the many genes mapped to 1B, *Yr15* and *Yr10* are located closest to *QYrco.wpg-1B*.*1*. *Yr15* has been linked to several markers that were mapped to 1B in this study, including *Xgwm413*, which was found to be 26.4 cM distal of *QYrco.wpg-1B.1* and was reported by Murphy et al. [59] to be completely linked with *Yr15*. *Yr15* confers resistance to all known races of stripe rust and therefore could not be present in the population because both Tres and Madsen are susceptible to at least one known race of stripe rust and are not known to carry *Yr15* [5] (Table 3). Therefore, neither parental line could be a donor of *Yr15*. *Yr10* is reported to be on 1BS at 1.2 cM from *Xpsp3000* [60]. *Xpsp3000* mapped (4.4 cM) proximal from the peak of Pst-100 and 2.0 cM distal from the peak of Pst-127, suggesting that this QTL may be an effect of *Yr10*. Although map position indicates this QTL could be *Yr10*, along with a similar virulence/avirulence formula (Table 3) other data suggest that it is not the case. In Tres, the absence of marker allele size for *Xgwm11* (linked to *Yr10*) and absence of linkage to *Rg1* (brown chaff color) both of which have been linked to *Yr10* [48], [61] suggests lack of *Yr10*. Additionally, the parents of Tres were not carriers of brown chaff color, further indicating Tres did not inherit *Yr10* (Dr. Robert Allen, personal communication, May 13, 2013). A close look at the pedigree of Tres does not indicate parentage containing Yr10, unlike Moro, which has brown chaff and whose parentage contains PI178383, the proposed donor of *Yr10* [60]. Further evidence that Tres does not carry *Yr10* is presented in Chen and Line [29], where the genes for Tres were investigated. After evaluation of F_2_ populations and chi-square tests for goodness of fit for stripe rust resistance, it was concluded that Tres and Moro contained different stripe rust genes [29]. This conclusion led to Moro and Tres being used as race differentials four and 14, respectively [5]. Thus, although *QYrco.wpg-1B*.*1* is in a similar location as *Yr10*, the fact that Tres does not carry *Rg1*, does not contain parentage which carries *Yr10*, and the work of Chen and Line [29] indicating Tres and Moro carry different genes, we suggest that *QYrco.wpg-1B*.*1* is different than *Yr10*, but in close proximity to this gene. Further allelism tests with Coda and Moro (apart from the Tres and Moro work published previously) are being conducted to confirm this.

**Table 3:** Observed and predicted virulence and avirulence formula of three races of stripe rust tests against the Brundage by Coda recombinant inbreed line population for seedling resistance.

	Gene^a^	PST-100	PST-114	PST-127
Brundage	Unknown	V	V	V
Coda	Unknown	A	A	A
*QYrco.wpg-1B.1*				
Chinese	*Yr1*(2A)	A	V	A
Tres	*YrTr1*(6D) *YrTr2*(3A)	A	V	A
Moro	*Yr10* (1B) *YrMor*(4B)	A	V	A
*QYrco.wpg-1B.2*				
Madsen	Unknown	A	A	V
Paha	*YrPa1 YrPa2 YrPa3*	A	A	V
Druchamp	*Yr3a*(1B) *YrD YrDru*(5B)	A	A	V

a Genes and virulence formula are based on Chen et al. 2010 and McIntosh et al. 2011 with chromosome in parentheses (if known).

The more probable identity of *QYrco.wpg-1B*.*1* would be one of the known genes in Tres. Earlier work [29] identified two dominant all-stage resistance genes, *YrTr1* and *YrTr2*, in Tres. Previous monosomic analysis placed *YrTr1* and *YrTr2* on chromosome 6D and 3A, respectively [62]. In our study, genes from Tres were not found on 6D nor 3A, but on 1B and 5B. Potentially, the work of Chen and Line [29] did not identify all the genes found in Tres. Another possibility is that the monosomic analysis performed by Chen et al. [45] incorrectly interpreted the results of the analysis and thus incorrectly placed the genes. Recent work done on Hessian fly resistance has proven monosomic analysis to give different results when compared to QTL analysis. The Hessian fly gene *H6* was originally mapped by Gallun and Patterson [63] to chromosome 5A through monosomic analysis. Subsequent genes, including *H3* and *H9*, were also mapped to 5A, either through monosoimc analysis or linkage to genes previously identified on 5A. Recently, three distinct F_2_ populations (‘Iris’ (PI 562615)×‘Newton’ (CItr 17715); Iris×‘Len’ (CItr 17790); and ‘Ella’ (CItr 17938)×Len) have provided evidence that *H9* resides on chromosome 1AS [64]–[65]. Further discussion by Liu et al. [64] and Kong et al. [65] disputed the location of many Hessian fly genes, including *H6* and *H3* due to misinterpretation of the monosomic mapping data. Thus, *QYrco.wpg-1B*.*1* is more likely *YrTr1* or *YrTr2* inherited from Tres if the monosomic analysis of Tres was similarly misinterpreted.

*QYrco.wpg-1B.2* also on chromosome 1B, shares a virulence formula with the stripe rust differentials ‘Chinese’, ‘Paha’ and ‘Druchamp’ (Table 3; [6]). Chinese is a known carrier of *Yr1* located on 2A [66] and is probably not this gene. The locations of Paha resistance genes are unknown. Durchamp carries *Yr3a* in addition to *YrD* and *YrDru* [5]. *Yr3a* is located on 1B, *YrDru* is on 5B and the location of *YrD* is unknown [67]–[68]. Furthermore, *Yr3a* is present in the cultivar Nord Desprez, which is in the background of Madsen, a parent of Coda [68]. Both Tres and Paha share Suwon 92 as a common parent, and thus a possible source of this resistance. Haplotype data and virulence data suggest that this QTL comes from the parent Madsen, and not Tres. Therefore, *QYrco.wpg-1B.2* may be an effect of *Yr3a*, although hard to confirm. Chen et al. [67]–[68] placed *Yr3a* on chromosome 1B based on monosomic analysis, so there is no genetic map published to compare chromosomal location. Further work will need to be done to confirm if *QYrco.wpg-1B.2* is *Yr3a* or a new gene.

Other potential genes that could be responsible for resistance found on chromosome 1B include: *Yr24/26/CH42*, *Yr9*, *YrExp1*, *Yr21*, *Yr29* and *YrAlp* (reviewed by [53]). The genes *Yr24/26/CH42* were reported to be the same gene and are present on 1BS in the region and were reported to be 2.3 cM away from *Xgwm11* and 2.5 cM from *Xgwm273* [50]. *Xgwm11* and *Xgwm273* were both mapped in this study however they were not present on the 1B linkage group. Furthermore, *Yr24/26/CH42* provides resistance to Pst-100, Pst-114, and Pst-127, and could not be present in either of the parental lines of Coda as Tres is susceptible to Pst-114 and Madsen susceptible to Pst-127. The genes *Yr9*, *YrExp1* and *Yr21* are susceptible to every race of stripe rust used in this study making it unlikely that either 1B QTL was an effect of these genes [6]. Furthermore as QTL on 1B confer all-stage resistance it is unlikely that adult plant resistance genes such as *Yr29* [52] or *YrAlp*, which is susceptible to PST-100 at the seedling stage, are responsible for observed effects at this locus [16].

QTL on chromosome 3B were all identified from field screening and appear to be adult plant resistance genes based on field reactions. Overall both of these QTL appear to be fairly stable across locations and years, with *QYrbr.wpg-3B.1* being observed in all locations, whereas *QYrbr.wpg-3B.2* showed greater variability. All of the QTL identified on 3B were inherited from Brundage and were of modest effect. Haplotype analysis suggests that this QTL was inherited from Stephens. Stephens has been characterized for stripe rust resistance QTL in two independent studies, neither of which found resistance QTL on chromosome 3B [18], [69]. Geneva was resistant to race PST-37, but susceptible to races PST-43, PST-45, and PST-78 in seedling tests, and also susceptible in field test in Mt Vernon in 2002 (Chen, unpublished data); and indicated to be susceptible to stripe rust in the cultivar registration [70]. Therefore, it is unlikely that Geneva contributed any detected QTL in Brundage. Several provisionally designated all-stage resistance genes have been identified from Stephens on 3B including *YrS* [67] and *YrSte2* [68]. However, both genes were identified based on seedling reaction making it unlikely that QTL on 3B was an effect of either of these genes. However, since we screened using different races than Chen et al. [68] there still may be a possibility that *QYrbr.wpg-3B.1* is *YrSte2*.

The *Sr2/Lr27/Yr30* adult plant rust resistance locus is on 3BS [71]–[72]. This presents the possibility that QTL on 3B are an effect of this locus. *Sr2* is known to be associated with pseudo-black chaff [73]. Neither Brundage, Stephens, nor the RIL population have been identified as having pseudo-black chaff and are susceptible to stem rust (data not shown). It could be that this linkage has been broken, but is very unlikely as previous fine mapping efforts have not been able to dissociate these two traits [74]. Pseudo-black chaff does not express in every environment and is controlled by modifier genes [75], so it could be that *Sr2/Yr30* is present, but expression of the pseudo-black chaff has not been visible or the associated modifier genes were not inherited. Consequently, the closest linked SSR marker to *Sr2* is *Xgwm533* [76], which was monomorphic in this population. The more diagnostic CAPS marker *csSr2* predicted Stephens to have *Sr2*, whereas the SSR marker *Xgwm533* showed a susceptible 155 bp allele and field data stem rust reaction of 60SMS [77]. Thus, Stephens may carry the *Sr2/Yr30* locus, but it is not diagnostic using *Xgwm533*. These data leave inconclusive evidence of whether the 3B QTL is the *Sr2/Yr30* complex or not. Many other QTL have been identified on chromosome 3BS, but due to different marker platforms, it is difficult to tell if these are in similar locations or not.

*QYrco.wpg-5B* identified on chromosome 5B appears to be an adult plant resistance gene. Overall this QTL was of similar effect to QTL identified on 3B and was present in most locations. Possible known genes at this locus include *Yr19* and *YrDru*. *Yr19* confers resistance to race PST-127 and *YrDru* confers seedling resistance to Races PST-100 and PST-114 [6]. As this QTL was not found to be associated with seedling resistance to these races it is unlikely that this QTL is an effect of either of these known genes. *YrExp1* is also located on 5B and confers adult plant resistance [17]. The relationship between *QYrco.wpg-5B* and *YrExp1* is unknown and warrants further investigation. The relationship of *QYrbr.wpg-5B* with *Yr47* has not been determined, as this gene has not been tested with any U.S. races [78]. *QYrbr.wpg-5B* was derived from Coda and both Madsen and Tres have the same haplotype, indicating both parents may carry this QTL. This requires further experiments as the stripe rust resistance of Madsen has yet to be mapped and it carries high levels of adult plant resistance to stripe rust.

A minor seedling reaction QTL was found on chromosome 5D and was inherited from Brundage. The only known stripe rust resistance genes on chromosome 5D include *YrDa2* from ‘Daws’ [11] and *Yr40* [79]. *Yr40* is an introgression from *Aegilops geniculata* that is effective against PST-100, making it unlikely that this QTL is related to *Yr40*. The origin of *QYrbr.wpg-5D* is interesting as the contributing parent Brundage was scored as susceptible to PST-114. In multiple tests performed by Dr. Chen (unpublished data), Brundage 96 was resistant or moderately resistant showing heterogeneous reactions to PST-114. Brundage 96 was a reselection out of Brundage for higher levels of stripe rust resistance; therefore, it is possible that there is heterogeneity or heterozygosity within Brundage for this QTL that was captured in the RIL population. This is further supported by several observations of high disease incidence in the population but low disease on Brundage in several locations (Figure S1).

Coda has shown a high level of durable resistance for many years. The results presented here show that this resistance was provided by two major QTL, both of which are likely all-stage resistance genes. Individually, these QTL provide moderate resistance to stripe rust. Together, *QYrco.wpg-1B.1* and *QYrco.wpg-1B.2* provide effective resistance and a significant reduction in IT and DS mean values capable of substantially reducing stripe rust incidence across years and locations. Based on race specificity and pedigree analysis it is possible that these QTL are effects of a resistance genes in Tres combined with the resistance genes from Madsen. Virulence on Tres resistance and *Yr3a* (potential gene in Madsen) was reported in 22 and 67% of isolates collected in the U.S. in 2006 respectively [6]. Although individually each one of these genes has been defeated, the complementary action of these two genes provides durable resistance in Coda. This resistance was further aided by several adult plant QTL. Individually, the adult plant resistance QTL demonstrated minor effect against mix field populations. Once combined with other QTL of similar effect, resistance increased significantly. Pyramiding of multiple stripe rust resistance genes can improve durability and efficacy of deployed resistance genes, as demonstrated by the strong durable resistance in Coda. Combining *QYrco.wpg-1B.1* and *QYrco.wpg-1B.2* with other resistance genes will prove to be an effective stripe rust resistance breeding strategy.

## Supporting Information

Figure S1 **RIL disease observations.** Mean values for: (**a**) stripe rust infection type (IT) and (**b**) disease severity (DS) relative area under the disease progress curve (rAUDPC) values for the resistant parent Coda, susceptible parent Brundage and the entire recombinant inbred line (RIL) population. Locations include Central Ferry, WA (CF); Mount Vernon, WA (MV); Spillman Farm Pullman, WA (PU); Whitlow Farm Pullman, WA (WL); and Parker Farm Moscow, ID (UI). Dates include 2006 (06), 2010 (10) and 2011 (11).
(DOCX)

Figure S2 **Full linkage map of significant linkage groups.** Full linkage maps of the four wheat chromosomes 1B, 3B, 5B, and 5D, which were associated with stripe rust infection type and disease severity values using QTL analysis in the Brundage by Coda recombinant inbred line population.
(DOCX)

Table S1 **Analysis of variance of infection type.** Values evaluated for replicate and genotype effect for each location. Heritability (H^2^) analysis shown in left two columns for each location as well as heritability evaluated across all locations used in this study. Locations include Central Ferry, WA (CF); Mount Vernon, WA (MV); Spillman Farm Pullman, WA (PU); Whitlow Farm Pullman, WA (WL); and Parker Farm Moscow, ID (UI). Dates include 2005–2006 (506), 2009–2010 (910) and 2010–2011 (1011).
(DOCX)

Table S2 **Analysis of variance of disease severity.** Values evaluated for replicate and genotype effect for each location. Heritability (H^2^) analysis shown in left two columns for each location as well as heritability evaluated across all locations used in this study. Locations include Central Ferry, WA (CF); Mount Vernon, WA (MV); Spillman Farm Pullman, WA (PU); Whitlow Farm Pullman, WA (WL); and Parker Farm Moscow, ID (UI). Dates include 2005–2006 (506), 2009–2010 (910) and 2010–2011 (1011).
(DOCX)
