# Supplementary material for: Mapping Stripe Rust Resistance in a BrundageXCoda Winter Wheat Recombinant Inbred Line Population
Source: PLoS One. 2014 Mar 18;9(3):e91758. doi: 10.1371/journal.pone.0091758 (PMC3958369; doi:10.1371/journal.pone.0091758)
Supplement: Figure S1 — RIL disease observations. Mean values for: (a) stripe rust infection type (IT) and (b) disease severity (DS) relative area under the disease progress curve (rAUDPC) values for the resistant parent Coda, susceptible parent Brundage and the entire recombinant inbred line (RIL) population. Locations include Central Ferry, WA (CF); Mount Vernon, WA (MV); Spillman Farm Pullman, WA (PU); Whitlow Farm Pullman, WA (WL); and Parker Farm Moscow, ID (UI). Dates include 2006 (06), 2010 (10) and 2011 (11). (DOCX) [file pone.0091758.s001.docx]

a

b

**Figure S1** Mean values for: (**a**) stripe rust infection type (IT) and (**b**) disease severity (DS) relative area under the disease progress curve (rAUDPC) values for the resistant parent Coda, susceptible parent Brundage and the entire recombinant inbred line (RIL) population. Locations include Central Ferry, WA (CF); Mount Vernon, WA (MV); Spillman Farm Pullman, WA (PU); Whitlow Farm Pullman, WA (WL); and Parker Farm Moscow, ID (UI). Dates include 2006 (06), 2010 (10) and 2011 (11)
