# Supplementary material for: Mapping Stripe Rust Resistance in a BrundageXCoda Winter Wheat Recombinant Inbred Line Population
Source: PLoS One. 2014 Mar 18;9(3):e91758. doi: 10.1371/journal.pone.0091758 (PMC3958369; doi:10.1371/journal.pone.0091758)
Supplement: Figure S2 — Full linkage map of significant linkage groups. Full linkage maps of the four wheat chromosomes 1B, 3B, 5B, and 5D, which were associated with stripe rust infection type and disease severity values using QTL analysis in the Brundage by Coda recombinant inbred line population. (DOCX) [file pone.0091758.s002.docx]

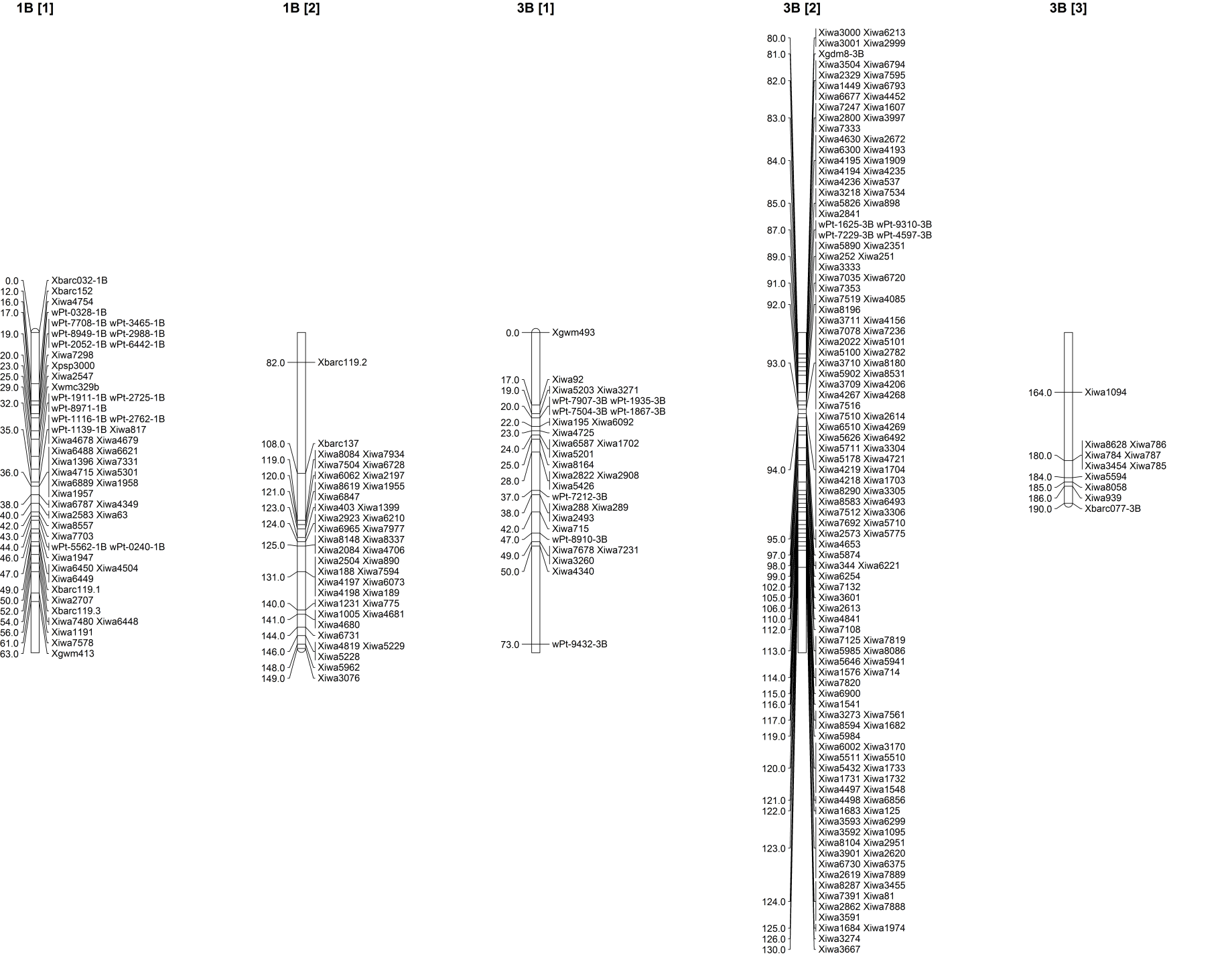


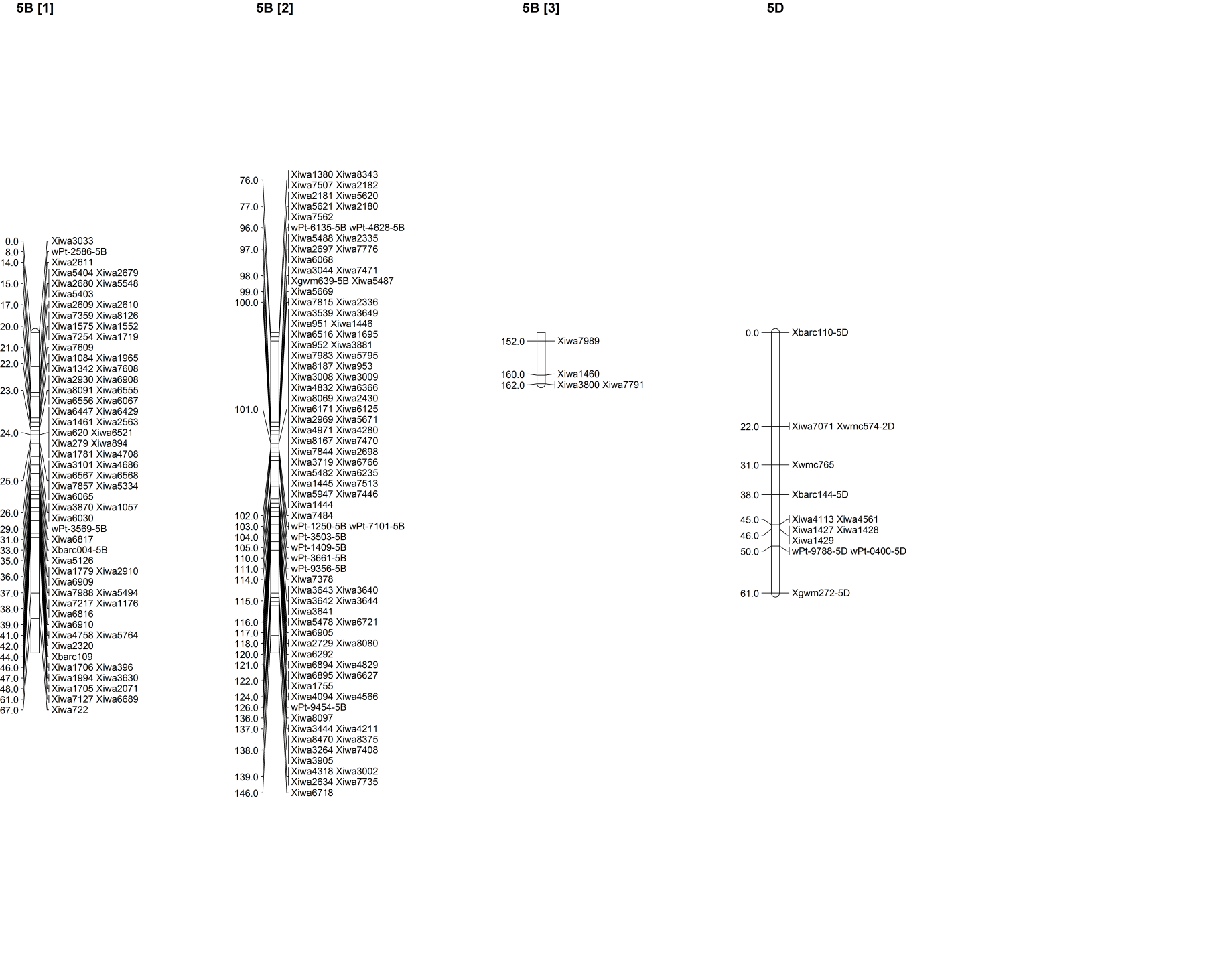


**Figure S2** Full linkage maps of the four wheat chromosomes 1B, 3B, 5B, and 5D, which were associated with stripe rust infection type and disease severity values using QTL analysis in the Brundage by Coda recombinant inbred line population.
