# Supplementary material for: Mapping Stripe Rust Resistance in a BrundageXCoda Winter Wheat Recombinant Inbred Line Population
Source: PLoS One. 2014 Mar 18;9(3):e91758. doi: 10.1371/journal.pone.0091758 (PMC3958369; doi:10.1371/journal.pone.0091758)
Supplement: Table S1 — Analysis of variance of infection type. Values evaluated for replicate and genotype effect for each location. Heritability (H2) analysis shown in left two columns for each location as well as heritability evaluated across all locations used in this study. Locations include Central Ferry, WA (CF); Mount Vernon, WA (MV); Spillman Farm Pullman, WA (PU); Whitlow Farm Pullman, WA (WL); and Parker Farm Moscow, ID (UI). Dates include 2005–2006 (506), 2009–2010 (910) and 2010–2011 (1011). (DOCX) [file pone.0091758.s003.docx]

**Table S1** Analysis of variance of infection type values evaluated for replicate and genotype effect for each location. Heritability (H^2^) analysis shown in left two columns for each location as well as heritability evaluated across all locations used in this study. Locations include Central Ferry, WA (CF); Mount Vernon, WA (MV); Spillman Farm Pullman, WA (PU); Whitlow Farm Pullman, WA (WL); and Parker Farm Moscow, ID (UI). Dates include 2005-2006 (506), 2009-2010 (910) and 2010-2011 (1011)

| Location | Effect | df | MS | F-value | P-value | R^2^ | Coefficient of variance | H^2^ | Standard error |
| --- | --- | --- | --- | --- | --- | --- | --- | --- | --- |
| cf10 | Replicate | 2 | 7.434 | 3.34 | 0.0362 | 0.754 | 44.902 | 0.633 | 0.0295 |
|  | Genotype | 267 | 14.033 | 6.30 | <.0001 |  |  |  |  |
|  | Error | 530 | 2.223 |  |  |  |  |  |  |
| ui11 | Replicate | 2 | 34.434 | 23.35 | <.0001 | 0.843 | 27.5 | 0.695 | 0.0345 |
|  | Genotype | 261 | 9.237 | 6.26 | <.0001 |  |  |  |  |
|  | Error | 307 | 1.474 |  |  |  |  |  |  |
| ui10 | Replicate | 1 | 3.1361 | 2.04 | 0.1548 | 0.826 | 119.7 | 0.654 | 0.0343 |
|  | Genotype | 267 | 7.3839 | 4.79 | <.0001 |  |  |  |  |
|  | Error | 267 | 1.5406 |  |  |  |  |  |  |
| mv06 | Unreplicated | |  |  |  |  |  |  |  |
| mv10 | Replicate | 2 | 4.685 | 4.78 | .0088 | 0.997 | 35.73 | 0.707 | 0.025 |
|  | Genotype | 267 | 8.061 | 8.22 | <.0001 |  |  |  |  |
|  | Error | 533 | 0.984 |  |  |  |  |  |  |
| pu11 | Replicate | 1 | 1.9104 | 2.12 | 0.146 | 0.898 | 20.18 | 0.707 | 0.025 |
|  | Genotype | 267 | 7.958 | 8.85 | <.0001 |  |  |  |  |
|  | Error | 267 | 0.899 |  |  |  |  |  |  |
| wl06 | Unreplicated | |  |  |  |  |  |  |  |
| wl10 | Replicate | 2 | 2.895 | 3.96 | 0.0197 | 0.883 | 30.1 | 0.818 | 0.017 |
|  | Genotype | 267 | 10.85 | 14.82 | <.0001 |  |  |  |  |
|  | Error | 534 | 0.731 |  |  |  |  |  |  |
| pu10 | Replicate | 2 | 5.846 | 1.18 | 0.3083 | 0.699 | 54.9 | 0.549 | 0.037 |
|  | Genotype | 267 | 5.797 | 2.43 | 0.043 |  |  |  |  |
|  | Error | 533 | 4.95 |  |  |  |  |  |  |
| Overall |  |  |  |  |  |  |  | 0.723 | 0.152 |
